# Correction: Bermudez-Lekerika et al. Sulfated Hydrogels as Primary Intervertebral Disc Cell Culture Systems. *Gels* 2024, *10*, 330

**DOI:** 10.3390/gels10100612

**Published:** 2024-09-24

**Authors:** Paola Bermudez-Lekerika, Katherine B. Crump, Karin Wuertz-Kozak, Christine L. Le Maitre, Benjamin Gantenbein

**Affiliations:** 1 Tissue Engineering for Orthopaedics and Mechanobiology, Bone & Joint Program, Department for BioMedical Research (DBMR), Medical Faculty, University of Bern, 3008 Bern, Switzerland; paola.bermudez@unibe.ch (P.B.-L.); katherine.crump@unibe.ch (K.B.C.); 2 Graduate School for Cellular and Biomedical Sciences (GCB), University of Bern, 3012 Bern, Switzerland; 3 Department of Biomedical Engineering, Rochester Institute of Technology, Rochester, NY 14623, USA; kwbme@rit.edu; 4 Spine Center, Schön Klinik München Harlaching Academic Teaching Hospital, Spine Research Institute, Paracelsus Private Medical University Salzburg (Austria), 81547 Munich, Germany; 5 Division of Clinical Medicine, School of Medicine and Population Health, University of Sheffield, Sheffield S10 2TN, UK; c.lemaitre@sheffield.ac.uk; 6 Inselspital, Department of Orthopedic Surgery & Traumatology, Medical Faculty, University of Bern, 3010 Bern, Switzerland

## Error in Figure

In the original publication [1], there was a mistake in Figure 5 regarding DNA amount units. According to the original publication, DNA amount was reported in µg/mL; however, the correct unit was ng/mL. After correction, the y axis titles in Figure 5 have now been modified to show values in ng/mL, as shown below in the corrected Figure 5.

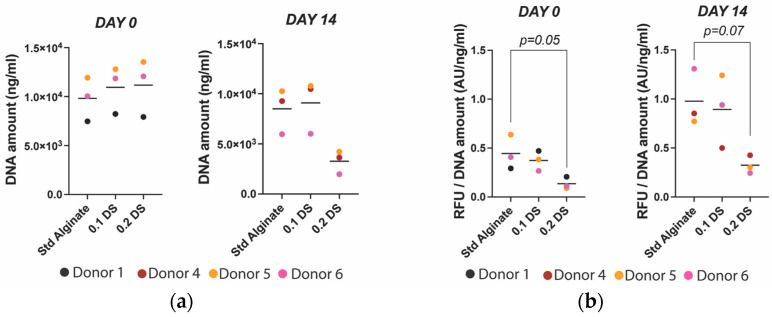



As a result of this correction to Figure 5, only DNA amount units have been corrected, maintaining the same DNA amount and metabolic activity proportional values.

The authors state that the scientific conclusions are unaffected. The Academic Editor approved this correction. The original publication has also been updated.
