# Supplementary material for: Sulfated Hydrogels as Primary Intervertebral Disc Cell Culture Systems
Source: Gels. 2024 May 14;10(5):330. doi: 10.3390/gels10050330 (PMC11121347; doi:10.3390/gels10050330)
Supplement: Supplementary file 1 [file gels-10-00330-s001.zip › Figure S1.pdf]

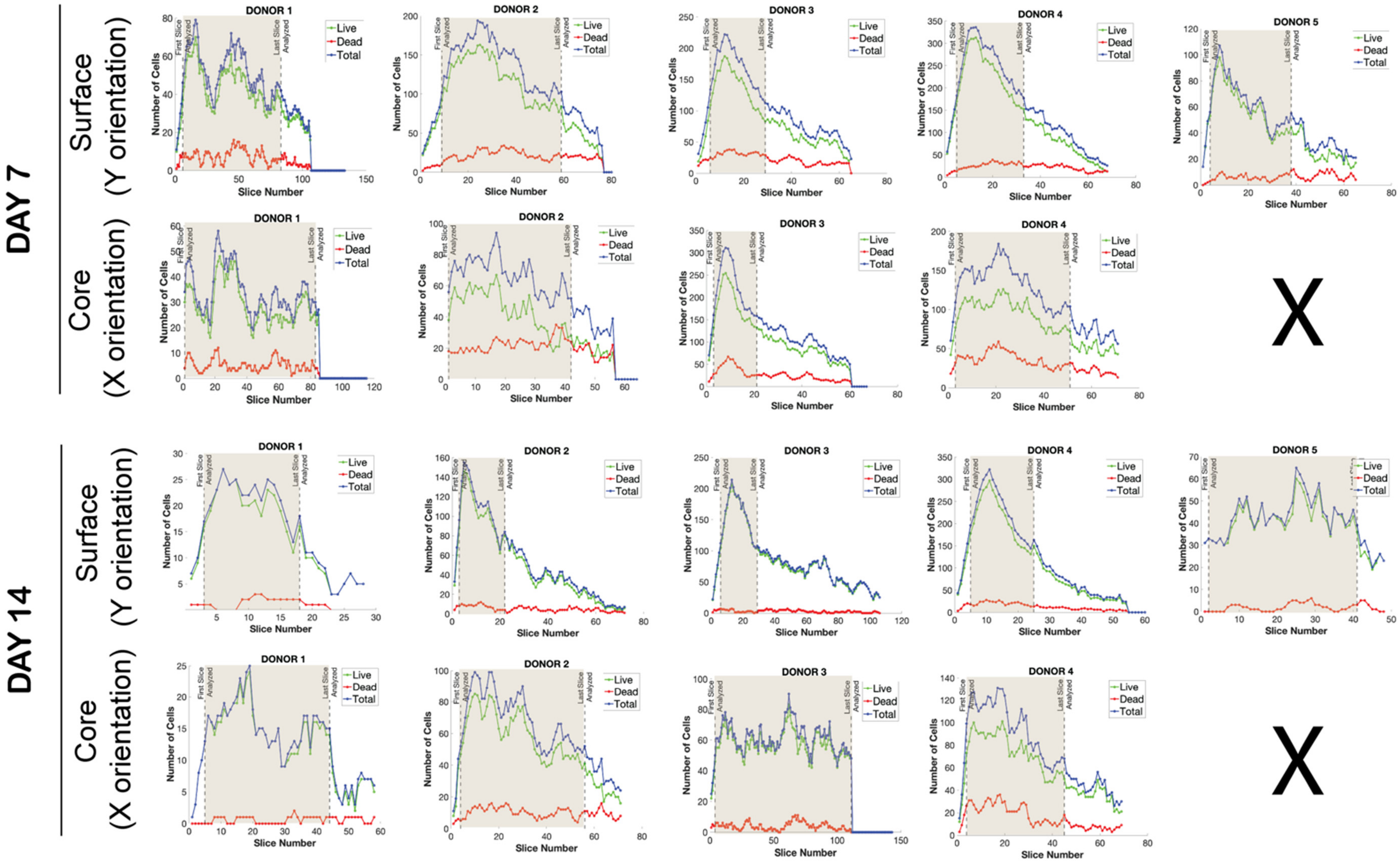

Figure S1. Collected and examined (shaded region) **c**onfocal slice stacks in the surface (Y orientation) and core (X orientation) of standard alginate carriers after seven and 14 days of culture. Green= Living cell amount; Red= Dead cell amount; Blue= Total cell amoun
