# Supplementary material for: Sulfated Hydrogels as Primary Intervertebral Disc Cell Culture Systems
Source: Gels. 2024 May 14;10(5):330. doi: 10.3390/gels10050330 (PMC11121347; doi:10.3390/gels10050330)
Supplement: Supplementary file 1 [file gels-10-00330-s001.zip › Table S1.pdf]

Table S1. Average density of human NP cells in Standard, 0.1% DS and 0.2% DS alginate.

Biomaterial	Location	Timepoint [Days]	Density Average [cells/ carrier]	± Standard Deviation
Standard Alginate	Surface	7	2.78 x10^5	1.70 x10^5
	Core	7	2.31 x10^5	1.69 x10^5
	Surface	14	2.22 x10^5	1.76 x10^5
	Core	14	1.23 x10^5	6.75 x10^5
0.1% DS Alginate	Surface	7	1.79 x10^5	9.03 x10^4
	Core	7	1.32 x10^5	3.60 x10^4
	Surface	14	1.54 x105	4.49 x10^4
	Core	14	1.24 x105	4.06 x10^4
0.2 % DS Alginate	Surface	7	7.13 x104	2.63 x10^4
	Surface	14	4.68 x104	6.45 x10^3
